# Editorial: Allergy in the Asia Pacific

**DOI:** 10.3389/falgy.2025.1567647

**Published:** 2025-02-21

**Authors:** Mongkhon Sompornrattanaphan, Agnes Sze-Yin Leung, Jane Chi Yan Wong, Philip Hei Li

**Affiliations:** ^1^ Division of Allergy and Clinical Immunology, Department of Medicine, Faculty of Medicine Siriraj Hospital, Mahidol University, Bangkok, Thailand; ^2^ Faculty of Medicine Siriraj Hospital, Center of Research Excellence in Allergy and Immunology, Mahidol University, Phuttamonthon, Thailand; ^3^ Department of Paediatrics, Faculty of Medicine, Prince of Wales Hospital, The Chinese University of Hong Kong, Hong Kong, Hong Kong SAR, China; ^4^ Hong Kong Hub of Paediatric Excellence (HOPE), The Chinese University of Hong Kong, Hong Kong, Hong Kong SAR, China; ^5^ Division of Rheumatology and Clinical Immunology, Department of Medicine, Queen Mary Hospital, The University of Hong Kong, Hong Kong, Hong Kong SAR, China; ^6^ Division of Rheumatology & Clinical Immunology, Department of Medicine, University of Hong Kong-Shenzhen Hospital, Shenzhen, Guangdong, China

**Keywords:** allergy, Asia Pacific, hypersensitivity, drug, urticaria, olfactory, chronic rhinosinusitis, angioedema

**Editorial on the Research Topic**
Allergy in the Asia Pacific

With approximately 4.7 billion people, or 60% of the global population, living in Asia and the Pacific, the region offers a unique perspective on the complexities of allergic disease. With diverse genetic backgrounds, cultural influences, environmental exposures, and healthcare systems, the region faces a rising prevalence of allergies driven by urbanization, pollution, and lifestyle changes, alongside significant variation in allergen sensitization patterns and prescribing practices (1–5). These factors not only pose significant public health challenges but also provide valuable opportunities to develop innovative, tailored approaches to allergy diagnosis and management. This research topic brings together impactful contributions that highlight drug allergy research, diagnostic advancements, and collaborative care pathways, all aimed at addressing the specific needs of the Asia-Pacific population.

Kan et al. explored the feasibility of an excipient allergy registry, focusing on polyethylene glycol (PEG) as a relevant example amidst global concerns about potential “allergies” to the COVID-19 vaccine. The authors highlighted a significant problem—the absence of a compulsory requirement to disclose the composition of pharmaceutical formulations. Their study disclosed that information about excipients was missing in more than 60% of drug formulations in Hong Kong, posing a substantial obstacle to precise diagnosis and patient safety. By advocating for universal legislative reform to mandate ingredient disclosure, the authors not only addressed a critical gap in allergy management but also laid the groundwork for improved global drug safety standards.

Building on the theme of innovation and precision, Mak et al. explored the utility of Sniffin' Sticks and the TIB Smell Identification Test in diagnosing olfactory dysfunction (OD) in Chinese patients with chronic rhinosinusitis (CRS). OD, a prevalent and disabling characteristic of CRS, significantly affects patients’ quality of life. Their findings demonstrated a strong correlation between these culturally adapted diagnostic tools, validating their use in this population. This study underscores the importance of tailoring diagnostic approaches to regional and cultural contexts, enabling precise and meaningful assessments in allergy-related conditions.

Li et al. further discussed the Hong Kong–Macau Severe Hives and Angioedema Referral Pathway (SHARP), a multidisciplinary initiative addressing severe chronic spontaneous urticaria (CSU) and angioedema. SHARP integrates allergists, dermatologists, and pharmacists into a streamlined referral system supported by evidence-based guidelines. This pathway emphasizes the use of biologics, such as omalizumab, for patients unresponsive to antihistamines. By fostering collaboration and optimizing care, SHARP serves as a model for improving outcomes in resource-limited healthcare settings, demonstrating how multidisciplinary care pathways can address the growing burden of immunologic and allergic diseases.

Another article included here is the mini-review by Mak et al. The authors identified beta-lactam and penicillin allergies in the Asia-Pacific region, uncovering key differences compared to Western populations. Their investigation uncovered a lower prevalence of genuine beta-lactam allergies in this area, a pattern likely shaped by genetic factors and prescription habits. Nevertheless, the prevalent misidentification of beta-lactam allergies continues to be a substantial hindrance to effective antibiotic stewardship. This study advocates for regional de-labeling initiatives and multidisciplinary strategies to reduce unnecessary antibiotic avoidance, improve clinical outcomes, and reduce healthcare costs.

These articles shed light on the unique challenges and opportunities of allergy care in the Asia-Pacific region and highlight the impact of regionally tailored, multidisciplinary approaches. By addressing key gaps in diagnosis, management, and policy, they offer valuable insights that extend beyond local contexts. As the global allergy community works to improve healthcare delivery and advance knowledge, the findings in this Research Topic lay a strong foundation for future innovation, collaboration, and practical solutions that will benefit both the Asia-Pacific region and the broader field of immunology and allergy.
